# Absolute SESAM characterization via polarization-resolved non-collinear equivalent time sampling

**DOI:** 10.1007/s00340-022-07751-9

**Published:** 2022-01-19

**Authors:** Alexander Nussbaum-Lapping, Christopher R. Phillips, Benjamin Willenberg, Justinas Pupeikis, Ursula Keller

**Affiliations:** Department of Physics, Institute for Quantum Electronics, ETH Zürich, Auguste-Piccard-Hof 1, 8093 Zurich, Switzerland

## Abstract

Semiconductor saturable absorber mirrors (SESAMs) have enabled a wide variety of modelocked laser systems, which makes measuring their nonlinear properties an important step in laser design. Here, we demonstrate complete characterization of SESAMs using an equivalent time sampling apparatus. The light source is a free-running dual-comb laser, which produces a pair of sub-150-fs modelocked laser outputs at 1051 nm from a single cavity. The average pulse repetition rate is 80.1 MHz, and the full time window is scanned at 240 Hz. Cross-correlation between the beams is used to calibrate the time axis of the measurements, and we use a non-collinear pump-probe geometry on the sample. The measurements enable fast and robust determination of all the nonlinear reflectivity and recovery time parameters of the devices from a single setup, and show good agreement with conventional nonlinear reflectivity measurements. We compare measurements to a rate equation model, showing good agreement up to high pulse fluence values and revealing that the samples tested exhibit a slightly slower recovery at higher fluence values. Lastly, we examine the polarization dependence of the reflectivity, revealing a reduced rollover if cross-polarized beams are used or if the sample is oriented optimally around the beam axis.

## Introduction

Over the past several decades, ultrafast laser technology has progressed from laboratory demonstrations to widespread use in industry and scientific research. A key part of this progression has been the development of passively modelocked lasers producing ultrashort pulses. The core component in these lasers is a mechanism providing an intensity-dependent reduction in the net cavity losses, thereby favoring pulsed operation over continuous-wave lasing. Saturable absorbers have proven to be highly successful for this purpose [1]. Early passive modelocking experiments used dye lasers, which led to pulse durations as short as 27 fs [2]. However, these were challenging systems to operate, limiting their use to research labs. A major advance was the development of the semiconductor saturable absorber mirror (SESAM) [3, 4], which enabled robust self-starting passive modelocking of solid-state lasers. This in turn led to a rapid proliferation of many kinds of lasers and applications [5]. The fundamental principle of a SESAM is the filling of states, analogous to the saturation of absorption in a two-level laser system [6]. When an ultrashort pulse of sufficient fluence impinges upon a SESAM, the available absorption states are saturated by the leading edge of the pulse, and the rest of the pulse experiences negligible absorption. Since this saturable absorption mechanism depends primarily on pulse fluence rather than intensity [7], SESAMs find application in generating pulses over a very wide range of durations from tens of femtoseconds in passively modelocked lasers [8] to longer pulses in passively Q-switched lasers [9].

In free-space laser oscillators (including solid-state lasers), the two dominant techniques used for ultrashort pulse generation are SESAM modelocking and Kerr-lens modelocking (KLM) [10]. Because of the near-instantaneous nature of the process, KLM has enabled the shortest pulses generated directly from a laser oscillator [8, 11]. Since its mechanism is an intensity-dependent change in the size of the laser cavity mode, which leads to either reduced losses through an aperture or better overlap with the pumped region in the laser medium, modelocking becomes strongly coupled to the stability of the cavity. Hence, such lasers usually require operation near the edge of cavity stability [12], making them more difficult and sensitive to align. In contrast, in SESAM-modelocked lasers, the cavity can be optimized for stability provided the fluence on the SESAM is within a suitable range, and it is straightforward to achieve self-starting operation. SESAMs can also be combined with KLM to obtain self-starting operation in combination with few-cycle pulses [8].

The above properties make SESAMs highly suited to industrial-grade laser operation over a wide range of parameters. For example, femtosecond SESAM-modelocked lasers have seen many advances in recent years including high repetition rates above 10 GHz [13], ultra-low-noise operation [14], monolithically integrated external cavity surface emitting lasers (MIXSELs) [15, 16], watt-level operation of Cr:ZnS lasers [17], single-cavity dual-comb generation [18–20], and very high average powers up to 350 W with sub-picosecond pulses [21].

Achieving ultrashort pulse operation places an important constraint on the SESAM: it must have an ultrafast recovery time. Fortunately, soliton modelocking allows for the saturable absorber recovery time to be several times the laser pulse duration [22]. Nonetheless, a recovery time of several picoseconds or less is still important for 100-fs class modelocked laser operation. In standard semiconductor materials, the recovery time is on the scale of nanoseconds [23], which is too long for ultrashort pulse generation. Introducing carrier trap states to the material leads to a much shorter recovery time [24]. With a sufficient trap density, the carriers decay on a picosecond timescale, thereby preventing lasing of continuous-wave light since the net-gain window is closed very soon after the ultrashort pulse. Multiple techniques have been developed for reducing recovery times in semiconductor devices including low-temperature growth [25, 26] and doping with ions. Such ions have been introduced in post-growth via ion implantation [27] or during the growth process itself [28]. Another strong motivation for III–V semiconductor devices with picosecond recovery times has been photoconductive antennas for generation and detection of terahertz radiation [29].

The resulting ultrafast recovery dynamics can be described on several different levels of abstraction. Modeling of ultrafast semiconductor dynamics is quite a challenging and computationally intensive task [30], especially when allowing for the defects introduced to obtain a fast recovery time. Therefore, for practical purposes, the recovery dynamics are often described by simplified rate equation models [7] in combination with a nonlinear loss term [31], or even more simply by a bi-exponential fit. While the ultrafast macroscopic polarization of the semiconductor material is not included in such models, the core behavior of absorption saturation and recovery can be captured empirically with sufficient fidelity to predict and design modelocked lasers. For this purpose, it is important to know the macroscopic parameters of the SESAM, namely its nonlinear reflectivity parameters [32] and recovery times [33].

To experimentally characterize the nonlinear reflectivity parameters, a pulse train is focused onto the sample, and the reflectivity is measured over a wide range of incident powers (a few orders of magnitude). Several techniques were explored for such measurements, ultimately leading to the approach of Maas et al*.* [34]. To determine the recovery times, a different setup based on pump-probe measurements is used: an intense pump pulse excites the SESAM and a much weaker probe pulse samples its response as a function of optical delay. It has been shown that performing such pump-probe measurements as a function of pump fluence can also yield precise information about the nonlinear reflectivity [35]. This is appealing since measuring all the relevant sample properties from one setup can simplify the otherwise time-consuming characterization process, and measuring the SESAM’s temporal response as a function of fluence reveals additional information about the sample.

A difficulty with standard pump-probe measurements is their slow and alignment-sensitive nature due to the need for a mechanical delay line. In cases requiring hundreds of picoseconds or more of optical delay, the large range required on the translation stage also becomes a problem since care must be taken to avoid loss of intensity calibration due to diffraction. These issues can be overcome by the principle of equivalent time sampling (ETS) [36, 37]. The resulting systems are also referred to as asynchronous optical sampling (ASOPS) systems [38]. Such systems are comprised of a pair of ultrafast lasers with slightly different pulse repetition rates. Consequently, each subsequent pair of pulses from the two lasers are delayed by a small amount of time equal to Δ*f*_rep_/*f*_rep_^2^, where Δ*f*_rep_ is the repetition rate difference and *f*_rep_ is the repetition rate [37]. Hence, rapid and long-range delay scanning is provided without any moving parts. Note that the spectrum of each modelocked laser has a frequency comb structure, which means that the modelocked laser pair corresponds to a dual optical frequency comb (dual-comb). Dual-comb sources have been widely explored for applications involving heterodyne measurements, for example Fourier transform spectroscopy [39, 40]. In contrast, here we perform nonlinear pump-probe measurements that are insensitive to the relative phase between the two beams.

Here, we demonstrate complete characterization of the nonlinear reflectivity and recovery time properties of SESAM samples using ETS, for the first time to the best of our knowledge. The new ETS setup, which is driven by a novel dual-comb laser [20], shows good agreement with nonlinear reflectivity measurements. In addition, our measurements provide a detailed picture of the ultrafast temporal SESAM response, including how the recovery dynamics vary as a function of pulse fluence due to the complicated underlying semiconductor dynamics. Moreover, we study the nonlinear reflectivity as a function of polarization, showing a strong dependence on the relative polarizations of the pump and probe and also on the rotation of the crystalline sample around the beam axis. Our approach shows that a single ETS setup driven by a free-running dual-comb laser is sufficient to perform full characterization of SESAM and other semiconductor samples at high speed and with sufficient accuracy for modelocked laser design and development.

The paper layout is as follows. We first review the key aspects of SESAM characterization and models in Sects. 2 and 3. Then, in Sect. 4, we present the experimental setups, including the steps taken to ensure high-precision, high-dynamic-range measurements. In Sect. 5, we present our main experimental results before concluding in Sect. 6.

## SESAM parameters and characteristics

**Fig. 1:**
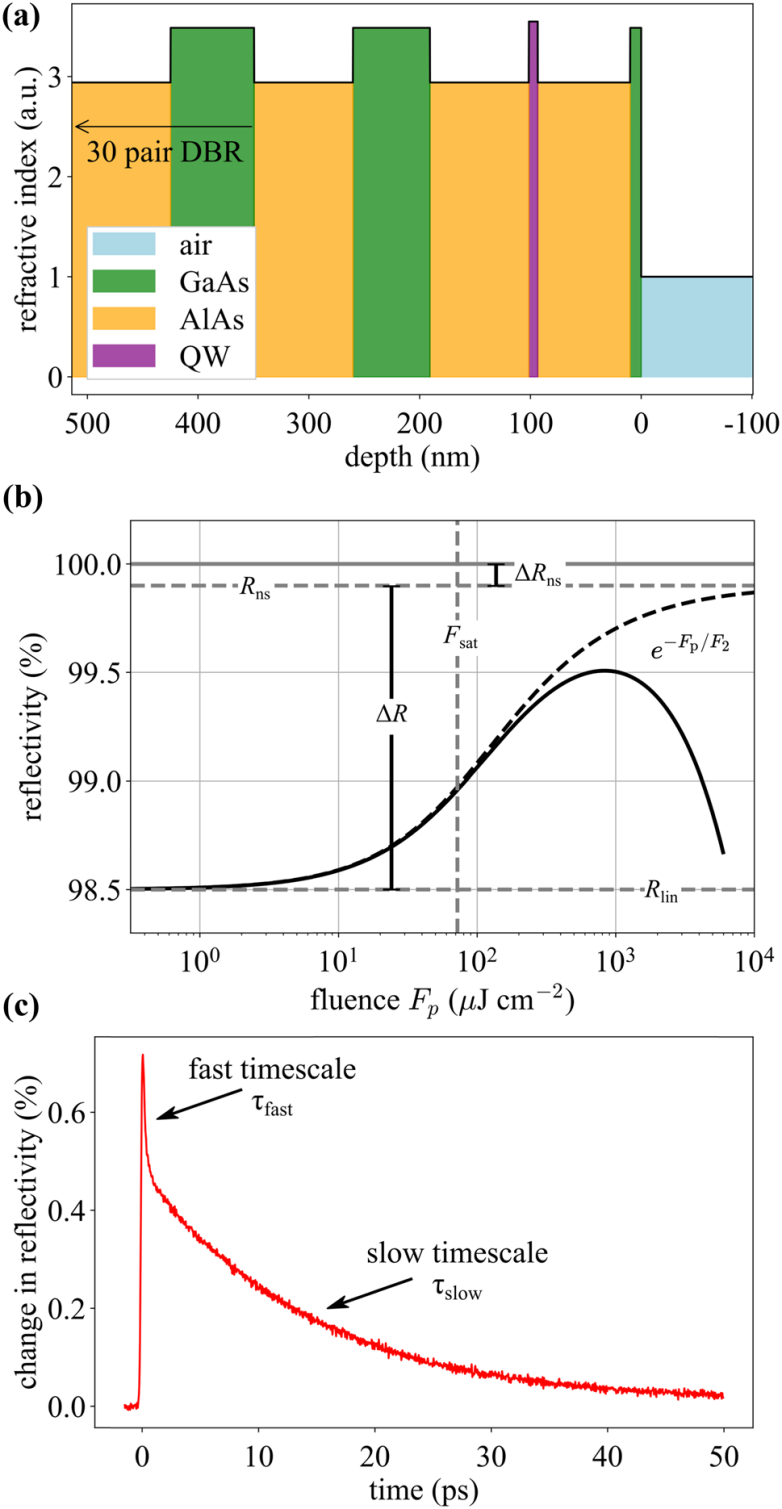
**a** A plot of the refractive index of the SESAM versus depth. The colors indicate the different layer compositions. **b** Semi-log plot of simulated reflectivity versus fluence with labels for the relevant parameters. *R*_lin_ = 98.5%, Δ*R* = 1.4%, *R*_ns_ = 99.9%, Δ*R*_ns_ = 1-*R*_ns_, *F*_sat_ = 72 μJ/cm^2^, *F*_2_ = 500 mJ cm^2^. **c** Experimentally measured temporal response of an example SESAM with > 15 ps recovery

In general, a SESAM is comprised of a semiconductor-based absorbing layer structure on top of a mirror structure. SESAMs have been explored in a variety of configurations [4]. An antiresonant design is often used since this provides broad bandwidth, low-dispersion, and high saturation fluence. Typically, the mirror is a distributed Bragg reflector (DBR), and there is one or more quantum well (QW)-based saturable absorber layers. Dielectric top coatings can be used to increase the saturation fluence and damage threshold [41], and substrate transfer and other techniques can be used for very high average power operation [42, 43]. For the SESAMs studied here, the DBR is based on an AlAs/GaAs layer stack, while the QWs are InGaAs layers embedded between non-absorbing AlAs layers. The QW composition and thickness is designed for operation at around 1050 nm. An example thin-film layer structure is shown in Fig. 1a. The fluence-dependent reflectivity is assumed to consist of three aspects:

1. The main saturable absorption mechanism,
2. An intensity-dependent loss term (also called inverse saturable absorption),
3. A fluence-independent offset in the reflectivity from 100%.

These characteristics are captured by the nonlinear reflectivity parameters as illustrated in Fig. 1b. The saturable absorption mechanism (1) is characterized by two parameters: a modulation depth Δ*R* which is the change in SESAM reflectivity after complete saturation of this mechanism (but neglecting the other mechanisms); and a saturation fluence *F*_sat_, which is the fluence at which a fraction 1/*e* of this loss has been saturated. The non-saturable reflectivity *R*_ns_ (mechanism (3)) characterizes an offset in the reflectivity from 100% that occurs due to any fluence-independent losses. The inverse saturable absorption (mechanism (2)) is characterized by the rollover fluence *F*_2_. This parameter incorporates any nonlinear loss effects which decrease the reflectivity of the SESAM such as two-photon absorption. Note that a finite *F*_2_ parameter implies a “rollover” in nonlinear reflectivity at high fluence. If two-photon absorption is the main process causing this rollover, then *F*_2_ is proportional to pulse duration (higher losses for shorter pulses). Consequently, when using shorter pulses, rollover occurs at lower fluence values and the maximum value of the nonlinear reflectivity curve is reduced. It is thus important to use a similar pulse duration to characterize the SESAM as the pulse duration that will be inside the laser cavity in the end.

Figure 1c shows an experimental measurement of the time-dependent reflectivity of an example SESAM. The fast decay time *τ*_fast_ is on the order of 100 fs and can be attributed to intraband thermalization [23, 44]. Interband recombination would lead to a much slower decay, on the timescale of nanoseconds. However, since the device has mid-gap traps, which in our case are introduced by low-temperature growth, the photo-excited carriers decay on a picosecond timescale. Note, for this figure, we chose a relatively slow SESAM sample to clearly illustrate the presence of fast (100-fs scale) and slow (in this example > 15 ps) recovery dynamics. The SESAMs we study in later sections have few-picosecond recovery times and are, therefore, better suited for modelocking in the 100-fs regime.

## Theory

The previous section provides an overview of the parameters that characterize a SESAM. This section gives an overview of the models needed to mathematically describe the experimental measurements.

The nonlinear reflectivity of the SESAM is usually derived via a rate equation model. Under the assumption that linear losses are not distributed through the length of the sample and that the response time is slow compared to the incident pulse duration (i.e., a slow saturable absorber), it is possible to derive the following expression for the nonlinear reflectivity *R* as a function of laser fluence *F* [7, 32]:

1 $$R\left( F \right) = R_{{{\text{ns}}}} \frac{{\ln \left[ {1 + \frac{{R_{{{\text{lin}}}} }}{{R_{{{\text{ns}}}} }}\left( {\exp \left( {F/F_{{{\text{sat}}}} } \right) - 1} \right)} \right]}}{{F/F_{{{\text{sat}}}} }}\exp \left( { - F/F_{2} } \right).$$

This formula applies at each point in space (i.e., it makes no assumption about the transverse beam profile), and the exponential rollover term (related to parameter *F*_2_) is added heuristically as a correction factor. The parameters are those discussed above and shown in Fig. 1b. By evaluating Eq. (1) as a function of transverse beam position, one can integrate the spatially varying reflectivity to find the pulse fluence-dependent reflectivity:

2 $$R_{{{\text{total}}}} \left( {F_{{\text{p}}} } \right) = \frac{{\int\nolimits_{0}^{\infty } {2\pi rF\left( r \right)R\left( {F\left( r \right)} \right){\text{d}}r} }}{{\int\nolimits_{0}^{\infty } {2\pi rF\left( r \right){\text{d}}r} }},$$

where *F*(*r*) is the local (transverse spatially varying) fluence profile of the pulse, and *F*_p_ is the average pulse fluence. The peak pulse fluence, i.e., the fluence at position *r* = 0, is *F*_pk_ = 2*F*_p_. In this paper, we follow the established convention of using *F*_p_ when plotting pulse fluence. For the usual case of a Gaussian beam, the radial integral can be simplified via a change of variables [32]:

3 $$R_{{{\text{Gauss}}}} \left( {F_{{\text{p}}} } \right) = \frac{1}{{F_{{\text{p}}} }}\int\limits_{0}^{{2F_{{\text{p}}} }} {R\left( F \right){\text{d}}F} .$$

Nonlinear reflectivity measurements directly sample Eq. (3) since a single pulse is focused on a sample and the directly reflected power is measured. However, for pump-probe measurements and especially ETS measurements the situation is slightly different. With ETS, the reflectivity of the probe beam is sampled as a function of pump-probe delay. Hence, for large delays (such that the SESAM has fully recovered from the pump-induced saturation), the measured voltage is proportional to Eq. (3) evaluated at the probe fluence. Conversely, at zero pulse delay, the measured voltage corresponds closely to Eq. (3) evaluated at the total fluence (pump plus probe), assuming equal size and spatial overlap of the two beams. Motivated by the structure of the measured signal, we define a normalized reflectivity $\overline{\Delta R} \sim \Delta R/R$, which is the change in reflectivity normalized to the reflectivity of the SESAM in the absence of a pump, as follows [19]:

4 $$\overline{\Delta R} \left( \tau \right) = \frac{{R_{{{\text{Gauss}}}} \left( {\tau ;F_{{\text{p, pump}}} ,F_{{\text{p, probe}}} } \right) - R_{{{\text{Gauss}}}} \left( {\infty ;F_{{\text{p, pump}}} ,F_{{\text{p, probe}}} } \right)}}{{R_{{{\text{Gauss}}}} \left( {F_{{\text{p, probe}}} } \right)}},$$

where *τ* corresponds to the relative delay between pump and probe. *R*_Gauss_(*τ*; *F*_p,pump_, *F*_p,probe_) is the reflectivity of the SESAM at delay *τ* in the presence of a pump and probe pulse. The second term in the numerator, with $\tau \to \infty$, corresponds to the limit of a large pump-probe delay. The normalization factor *R*_Gauss_(*F*_p,probe_) is the reflectivity of the SESAM due to the probe only, that is, with the pump beam physically blocked.

Since the signal is a change in reflectivity normalized to the reflectivity at low-fluence, we sample *R*_lin_/*R*_ns_ rather than *R*_lin_ and *R*_ns_ individually. A separate measurement of *R*_lin_ would be needed to rescale the measurement to obtain Δ*R* directly. For SESAMs with low losses and low modulation depth, such as those we use, this is a minor correction. Note also that although the single-pulse measurements and the ETS measurements sample *R*_Gauss_ in similar ways, there are some subtle considerations especially with respect to the rollover behavior of the two types of measurements, as shown in Sect. 5.3.

There have been different techniques to heuristically capture the time constants typically seen in the SESAM response. For example, the time-dependent losses *q*(*t*) of the SESAM can be approximated by a standard saturation formula [45]. In [46], a pair of such differential equations was used, one for each time constant:

5 $$\frac{{{\text{d}}q_{{\text{j}}} \left( t \right)}}{{{\text{d}}t}} = - \frac{{q_{{\text{j}}} \left( t \right) - q_{{\text{0,j}}} }}{{\tau_{{\text{j}}} }} - q_{{\text{j}}} \left( t \right)\frac{I\left( t \right)}{{F_{{{\text{sat}}}} }},$$

for $j \in \left\{ {\text{slow, fast}} \right\}$ and where *τ*_slow_ and *τ*_fast_ are the fast and slow recovery time, respectively. Here, *q*_0,slow_ and *q*_0,fast_ are the electric field absorption coefficients associated with the slow and fast recovery times, respectively, *I*(*t*) is the time-dependent optical intensity (the sum of pump and probe intensities), and *F*_sat_ is the saturation fluence of the SESAM. To use this model to calculate the probe reflectivity as a function of pump-probe delay *τ*, we first solve Eq. (5) using the pump *I*_pump_ and probe intensities *I*_probe_, i.e., *I*(*t*) = *I*_pump_(t) + *I*_probe_(*t*-*τ*), thereby finding the total SESAM losses *q*(*t*) = *q*_slow_(*t*) + *q*_fast_(*t*). The resulting probe reflectivity *R*_probe_, assuming small losses, is then given by

6 $$R_{{{\text{probe}}}} \left( \tau \right) = \frac{{\int_{ - \infty }^{\infty } {I_{{{\text{probe}}}} \left( {t^{\prime} - \tau } \right)\left[ {1 - 2q\left( {t^{\prime} - \tau } \right)} \right]\exp \left( { - \frac{{I_{{{\text{probe}}}} \left( {t^{\prime} - \tau } \right) + I_{{{\text{pump}}}} \left( {t^{\prime}} \right)}}{{I_{2} }}} \right)\text{d}t^{\prime}} }}{{\int_{ - \infty }^{\infty } {I_{{{\text{probe}}}} \left( {t^{\prime}} \right)\text{d}t^{\prime}} }},$$

where *I*_2_ is the intensity related to *F*_2_ with $I_{2} \approx 0.59F_{2} /\tau_{{{\text{FWHM}}}}$ for pulse duration τ_FWHM_. A less computationally intensive model is that of a bi-temporal exponential decay for Δ*R*(*t*):

7 $$\Delta R\left( t \right) = Ae^{{ - t/\tau_{{{\text{slow}}}} }} + \left( {1 - A} \right)e^{{ - t/\tau_{{{\text{fast}}}} }} ,$$

where *A* is the weight of the slow part of the response. This type of decay occurs once the pump intensity is negligible, which requires waiting for several pulse lengths such that the term proportional to *I*(*t*) in Eq. (5) becomes negligible. Equation (7) can be useful to estimate the slow recovery time constant with a simpler calculation compared to Eq. (5).

## SESAM characterization experiments

Next, we present our measurement setups used for SESAM characterization. Usually, to fully characterize SESAMs, one uses two measurement techniques. A single-pulse nonlinear reflectivity measurement is performed to determine the nonlinear reflectivity parameters and a pump-probe measurement is performed to obtain the temporal characteristics. Here, we replace the traditional pump-probe setup with an ETS setup based on a free-running dual-comb laser, and we show how this yields results largely consistent with conventional single-pulse nonlinear reflectivity measurements. In the following subsections, we first discuss the nonlinear reflectivity measurement setup based on the approach of [34]. Then, in Sects. 4.2 and 4.3, we introduce the equivalent time sampling setup and how it is used to enable high-precision measurements.

### Single-pulse nonlinear reflectivity

**Fig. 2:**
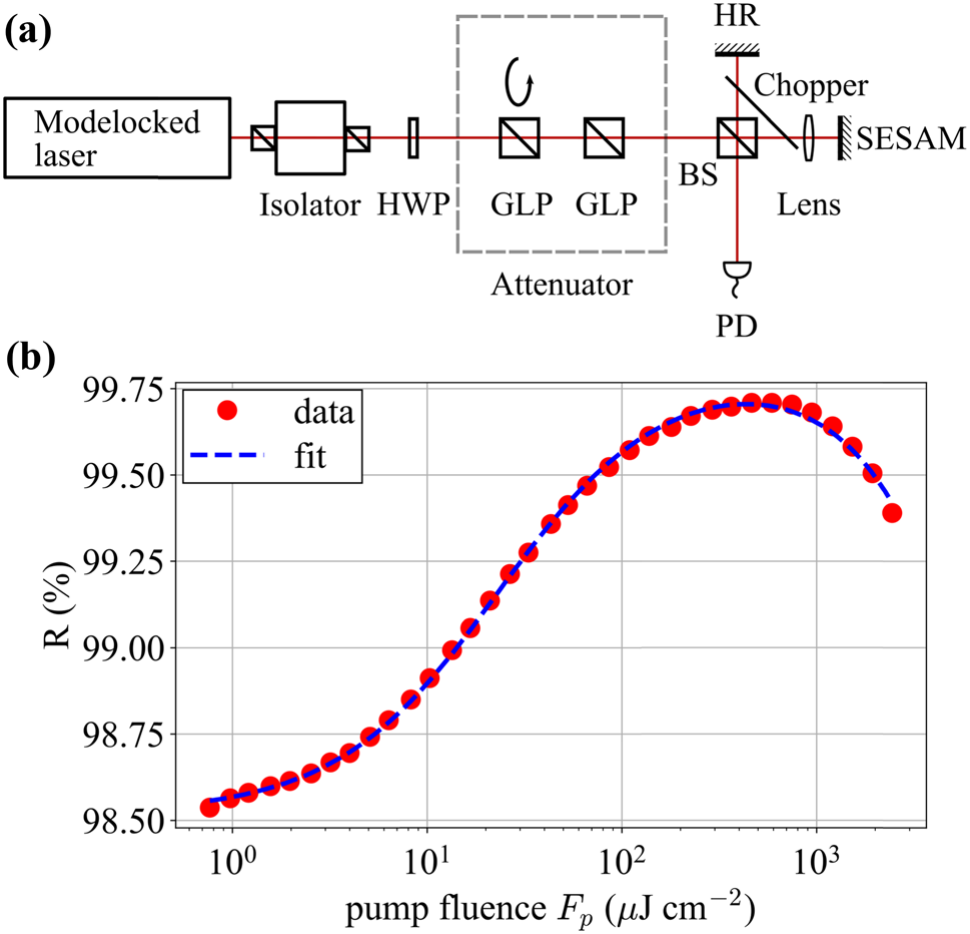
**a** Single-pulse nonlinear reflectivity measurement setup. **b** Example trace obtained from measurement. The red dots represent the data and the blue dashed line is a fit through the data using the model equation. The maximum power reaching the sample was 659 mW. *HWP* half-wave plate, *GLP* Glan-Laser polarizer, *BS* beam splitter, *HR* highly reflective mirror, *PD* integrating-sphere photodiode

An illustration of our single-pulse nonlinear reflectivity measurement setup, based on the approach of [34], is shown in Fig. 2a. An isolator is used to ensure no back reflected beams cause feedback into the laser. Next, a high-extinction-ratio Glan-Laser polarizer (GL10-B, Thorlabs Inc.) pair, the first of which is rotatable, is used as an attenuator and a half-wave plate is used to match the input beam’s polarization to that of the second polarizer. After this attenuator, the beam is directed to a 50:50 non-polarizing beam splitter cube to generate two beams. One is used as a reference beam and is back reflected with a highly reflective (HR) reference mirror. The other is focused with a 15-mm aspherical lens onto the SESAM under test. The reflection from the HR and the SESAM are combined in the 50:50 beam splitter and are ensured to be collinear before being detected by an integrating-sphere photodiode. It should be noted that isolators can introduce significant group delay dispersion that stretch the pulses. Therefore, we measured the pulse duration after the isolator with a frequency resolved optical gating (FROG) apparatus since SESAM fit parameters, especially *F*_2_, should be recorded along with the pulse duration at which they were measured; we measure a pulse duration of 183 fs at the sample position.

As described in [34] and shown in Fig. 2a, a chopper is placed such that its rotation axis lies above the plane of the beams. This allows for four states to be detected: (1) no beams (i.e., background); (2) only the reference beam; (3) both beams; and (4) only the SESAM reflected beam. By subtracting the background (state 1) from both of the signals where only one beam is detected (states 2 and 4) and taking their ratio, one can obtain the reflectivity of the SESAM. The absolute reflectivity is calibrated by measuring a highly reflective dielectric mirror at the same position. By measuring the sample at different attenuation levels, which in turn means different fluences, one can record a trace as seen in Fig. 2b. By fitting this fluence-dependent reflectivity data to Eq. (3), one can retrieve the nonlinear reflectivity parameters.

### Equivalent time sampling method

**Fig. 3:**
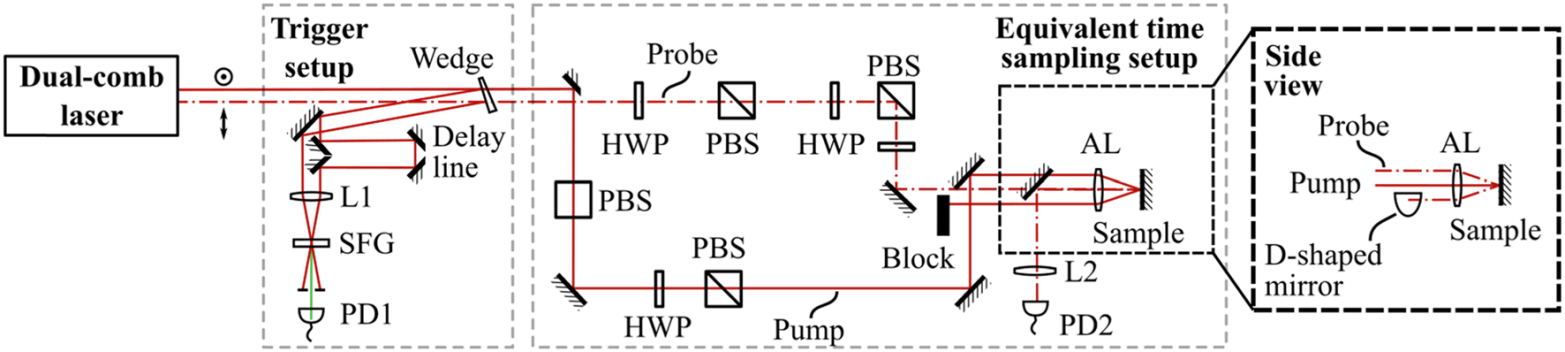
Schematic of the equivalent time sampling setup consisting of the dual-comb laser, the SFG-based trigger setup, and the non-collinear pump-probe setup. Right: side view of the non-collinear setup. The probe enters in from the top, is reflected, and exits at the bottom of the aspheric lens. A D-shaped mirror ensures that only the probe beam is detected by PD2. The reflected pump is blocked by a beam block. The time traces from the photodiode are digitized by a data acquisition card (PXI-5122, National Instruments corp.). SFG: type-I sum-frequency generation, L1: 100 mm focal length lens, AL: 50 mm focal length aspheric lens, L2: 200 mm focal length lens, PD1 amplified photodiode (PDA55, Thorlabs Inc.), PD2 amplified photodiode, HWP zero-order half-wave plate, PBS polarizing beam splitter (note that the first PBS in the pump path is rotated by 90° as the beam is vertically polarized.)

Our dual-comb ETS setup is depicted in Fig. 3. The laser system is the one recently presented in [20] which can deliver up to 1.8 W average power per comb with sub-150-fs pulse duration. The laser has a repetition rate of 80.1 MHz, and flexible repetition rate difference which was set to 240 Hz. The laser produces two frequency combs from a single cavity using the principle of birefringent multiplexing [18]. This approach allows for a convenient, simple, low-noise, and fully free-running laser pair with less complexity than standard ASOPS systems (which use two modelocked lasers). The automatically swept delay between the two pulse trains replaces the mechanical delay stage of traditional pump-probe setups. One of the two output beams (the one with vertical polarization at the output of the laser) is used as a strong pump pulse to excite a response in the SESAM, and the other output beam (with horizontal output polarization) is attenuated and used to probe the reflectivity. With a repetition rate difference of 240 Hz, a pulse-to-pulse time step of Δ*f*_rep_/*f*_rep_^2^ = 37.5 fs is obtained, and after a time of 1/Δ*f*_rep_ = 4.17 ms a full delay range of 1/*f*_rep_ = 12.5 ns has been scanned. Hence, we map the ultrashort timescale of the SESAM’s response to longer timescales which are easily measured by an oscilloscope. The scale factor between the measurement time and the response time is $$\Delta f_{{{\text{rep}}}} /f_{{{\text{rep}}}} \approx$$$$3 \cdot 10^{ - 6}$$.

Before the pump-probe part of the setup, we perform cross-correlation between the two combs to generate a trigger signal. As illustrated in Fig. 3, one of the beams of the cross-polarized output beam pair from the laser passes through a delay line before the two beams are made parallel. These beams are sampled with a wedged window and focused with a 100-mm lens into a 1.8-mm-long BBO crystal cut for Type-I sum-frequency generation. The beams cross in the BBO crystal, yielding a cross-correlation signal detected on a photodiode. This signal is used to trigger data acquisition when performing averaging on an oscilloscope/data acquisition card. In addition, it is used to track and account for any fluctuations of Δ*f*_rep_.

The main beams continue to the pump-probe setup where they are first separated into two distinct beam paths using a D-shaped mirror. Each path has a zero-order half-wave plate and polarizing beam splitter (PBS; PBS123, Thorlabs Inc.) for variable attenuation of the beams. The first PBS in the probe path is used to ensure that the polarization is well defined before being attenuated, as this increases the dynamic range of the measurement. The HWP in the pump beam path is mounted in a motorized mount (K10CR1/M, Thorlabs Inc.) so as to automate the measurement process. For the probe beam, there is a second HWP + PBS combination to reach the low probe fluence necessary for the measurements while still maintaining a clean polarization state. To allow for the pump and probe to be co- or cross-polarized a third zero-order HWP is included in the probe path after the second polarizing beam splitter cube. After attenuation, the beams are then non-collinearly focused onto the sample using an aspheric lens with a 50-mm focal length (AL2550-B, Thorlabs Inc.). The laser is operated in a configuration where it produces 141-fs pulses, and up to 1.16-W is available at the sample position. This maximum power would correspond to a fluence of *F*_p_ = 3.24 mJ/cm^2^ which is higher than the damage threshold of the SESAMs.

**Fig. 4:**
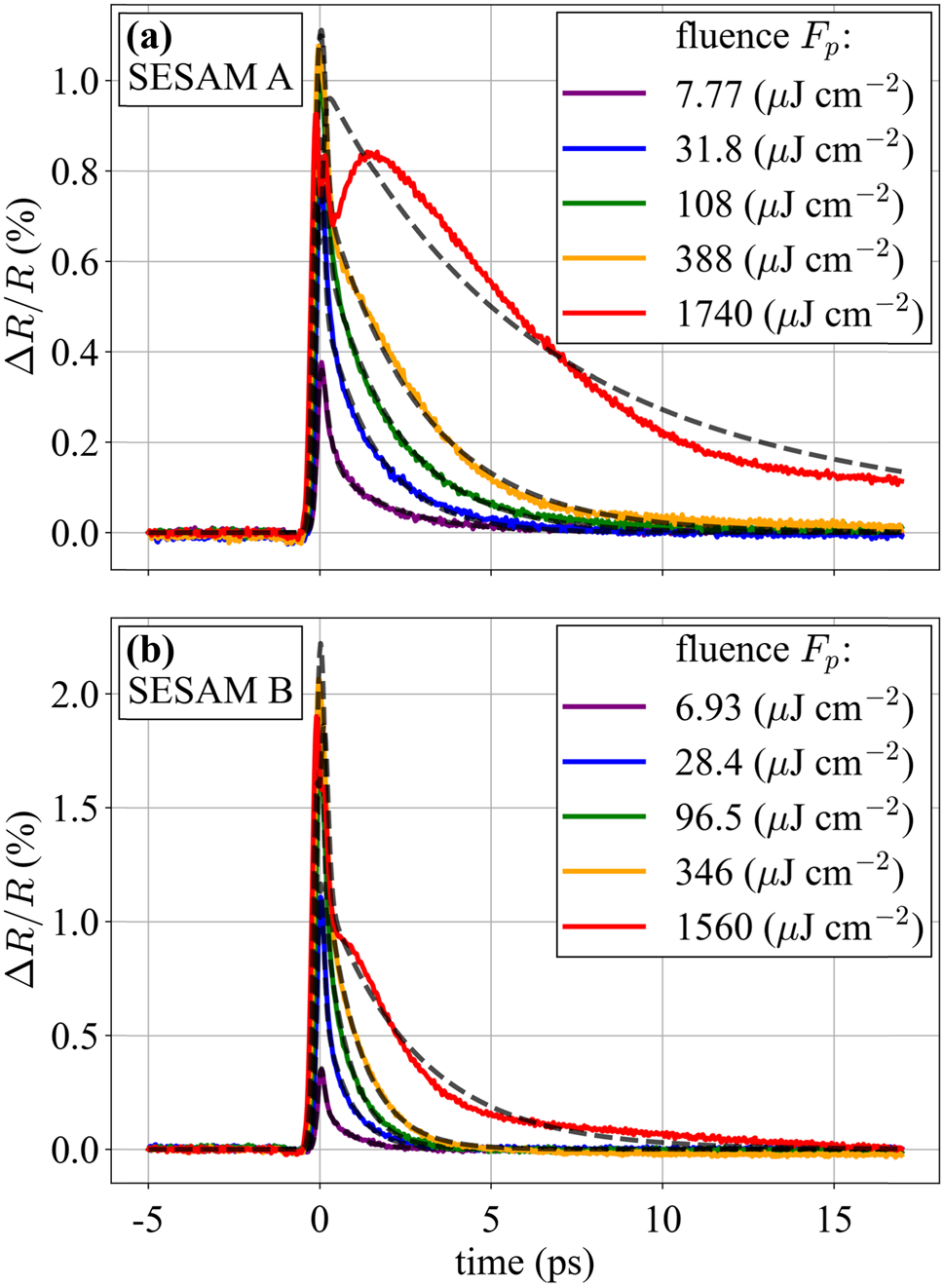
ETS time traces for two different samples at different fluences with a full fit of the trace (dashed lines). **a** A sample with a slower response and **b** a sample with sub-picosecond recovery. For both traces, time zero corresponds to the time of the trigger event. This condition was achieved by adjusting the delay line in the “trigger setup” section of Fig. 3

Since the probe is attenuated to a small fraction of the pump power, scattering or leakage of pump power onto the photodiode PD2 can potentially degrade the detected signal. To mitigate this issue, we use an out-of-plane focusing geometry: one beam is displaced vertically (the probe), while the other is displaced horizontally (the pump). Due to the non-collinear setup, the reflected probe from the sample is able to be separated with a D-shaped mirror. The probe beam is then focused onto an amplified InGaAs photodiode (PD) with a 200-mm lens. As the PD is fast enough to resolve the repetition rate of the dual-comb laser, the RF signal from the PD is filtered with a 30-MHz low-pass filter (BLP-30+, Mini-Circuits). Two photodiodes are used for the various measurements: for Figs. 4, 6 and 7, we use PDA10CF (Thorlabs Inc.) and a probe power of 132 μW; for Fig. 8 and 9, we use PDA10D2 (Thorlabs Inc.) and a probe power of 715 μW.

### Calibration for precision measurements

We observe a small cross-talk between the combs for delays corresponding to the time when the pulses meet in the cavity. To avoid any influence of this on the measurements, the difference in the path lengths between the oscillator output position and the sample position for the two combs is adjusted such that the condition when the pump and probe are temporally overlapped inside the cavity corresponds to slightly negative pump-probe delay times on the sample.

Various steps are included in the measurement procedure to ensure high repeatability and precision. First, a measurement where the pump and probe are blocked is taken to account for the background light. A measurement is taken with the pump blocked to find a voltage proportional to the probe-only reflectivity that is used in Eq. (4). The ETS traces are then acquired with AC coupling and any remaining offset from zero voltage at large delay is subtracted from the trace in a data processing step. Care is taken to avoid clipping of the probe beam reflected from the SESAM since this could lead to errors in the inferred reflectivity. In addition, stray pump light must be kept under control so as to not saturate the photodiode. Note that there is no HR mirror used as a calibration, and thus the non-saturable reflectivity *R*_ns_ cannot be extracted as in [35]. However, because we use SESAMs optimized for solid-state lasers which have *R*_ns_ close to 100%, as confirmed by nonlinear reflectivity measurements, the extracted quantity (*R*_ns_ − *R*_lin_)/*R*_ns_ is very close to the desired quantity of Δ*R* = *R*_ns_-*R*_lin_. In cases where the SESAM has high losses, *R*_lin_ in Eq. (1) could be calibrated by an HR reference sample or by a linear spectrophotometry measurement.

A critical point for extracting nonlinear reflectivity information is that the zero-delay point of the ETS trace must be known accurately, to a precision much shorter than the pulse duration [35]. Therefore, an ETS trace of a semiconductor DBR mirror is taken to calibrate for the zero-delay time, as the dip in the DBR trace corresponds to two-photon absorption which is assumed to be instantaneous for our purposes. This measurement provides the pump-probe zero-delay time relative to the trigger signal. To ensure this point is as stable as possible, the difference in path length between the two beams in the trigger setup has to match that of the ETS setup. We do this by delaying the vertically polarized beam in the trigger setup such that the zero-delay point, i.e., the TPA dip of the DBR, coincides with the trigger signal on the oscilloscope. As the trigger and signal occur at the same time, the zero-delay time of the pump-probe signal is less sensitive to small drifts in the repetition rate difference that can occur in a free-running dual-comb laser. This step could be avoided by tracking changes in Δ*f*_rep_, or implementing a slow Δ*f*_rep_ stabilization feedback loop. However, even without tracking Δ*f*_rep_, as the measurement is performed over a short period of time, the very small drifts found in [20] effect the measurement negligibly.

Since the setup uses a non-collinear geometry, we are not probing/pumping at zero angle of incidence (AOI), unlike in the single-pulse nonlinear reflectivity measurements. Therefore, care must be taken that the angle of incidence is sufficiently small to be representative of the AOI = 0° case. Two focusing configurations were tested: one with a 20-mm and another with a 50-mm focal length aspherical lens. The angle of incidence due to the focusing lens for the two setups is 25° and 10°, respectively. It was found that the measurement with the latter configuration showed slightly better agreement with the single-pulse measurement. This is consistent with the fact that the AOI can influence the SESAM’s linear and nonlinear reflectivity characteristics. All the measurements we present here used the 50-mm focal length configuration.

## Complete SESAM characterization

In this section, we present measurements on SESAM samples grown by molecular beam epitaxy (MBE) at the FIRST clean-room facility of ETH Zurich. The InGaAs QW layers were grown at low temperature. We selected two representative samples to study: one with a single QW design (sample A), the other with a three QW design (sample B). The layer structure of sample A was illustrated in Fig. 1a.

### Dynamic characteristics

The processed ETS traces at selected values of the pump fluence are shown in Fig. 4. The chosen fluence values show the transition from low saturation to a rollover regime. In this rollover regime, the simple differential equation model of Eq. (5) no longer captures the SESAM’s behavior, as seen for example in the 1740 μJ/cm^2^ curve in Fig. 4a. Even at lower fluences where it works well, inspection of the curves shows that their shape changes, so it is to be expected that the recovery times of the devices have some dependence on the fluence.

To quantify this behavior, a fitting procedure was performed on the measured data using the models discussed in Sect. 2 as follows. First, the measured reflectivity at zero delay as a function of pump fluence is compared with the ETS-augmented nonlinear reflectivity model of Eq. (4). This first fit is used to determine the nonlinear reflectivity parameters of the device (the parameters of Eq. (4) excluding the non-saturable losses). Second, the measured reflectivity versus time for a particular fluence is compared with the time-dependent probe reflectivity predicted by Eq. (6). This second fit is used to determine the recovery time values (the parameters of Eq. (5)), and is done separately for each value of the pump fluence. Both fits use a least-squares approach. The first fit assumes a Gaussian beam profile to determine the nonlinear parameters as accurately as possible. The second fit assumes a flat-top beam shape to reduce the computational requirements.

The simulated probe reflectivity using the output parameters from the fitting procedure are shown as dashed lines in Fig. 4. The model shows good agreement with the data up to fluences many times the saturation fluence, including values relevant for modelocked laser operation.

**Fig. 5:**
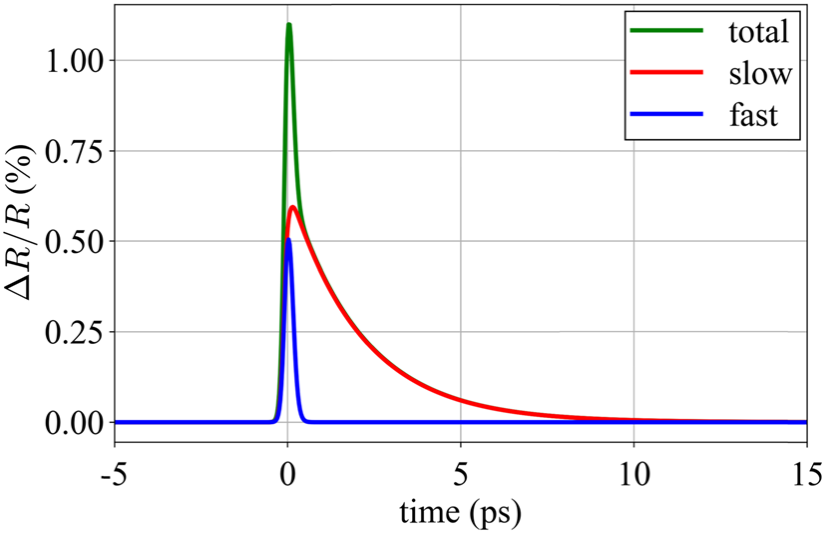
Example numerical fit or the recovery parameters. The individual contributions of the fast and slow parts are shown, along with the total response. This example corresponds to SESAM A with a pump fluence *F*_p_ = 108 μJ/cm^2^

To gain further insights into the predictions of the model, the two individual response terms (fast and slow) are shown in Fig. 5 for one example case. For this example, the contribution of the fast and slow parts of the response is comparable. Specifically, the relative reflectivity at zero-delay time due to only the slow response divided by the total response is approximately 52%. This fraction is used to determine the relative contribution of the slow part of the response. In addition, the fast part of the recovery closely resembles the pump pulse shape itself because the fitted recovery time is only on the order of 60 fs. Since this value is much less than the pulse duration of 141 fs it should not be interpreted as the true time constant, but rather that the femtosecond dynamics of the SESAM are faster than the resolution of the measurement. Measuring the fast recovery time more precisely would require shorter pump pulses, but for modelocking design purposes, the fit already provides sufficient information.

**Fig. 6:**
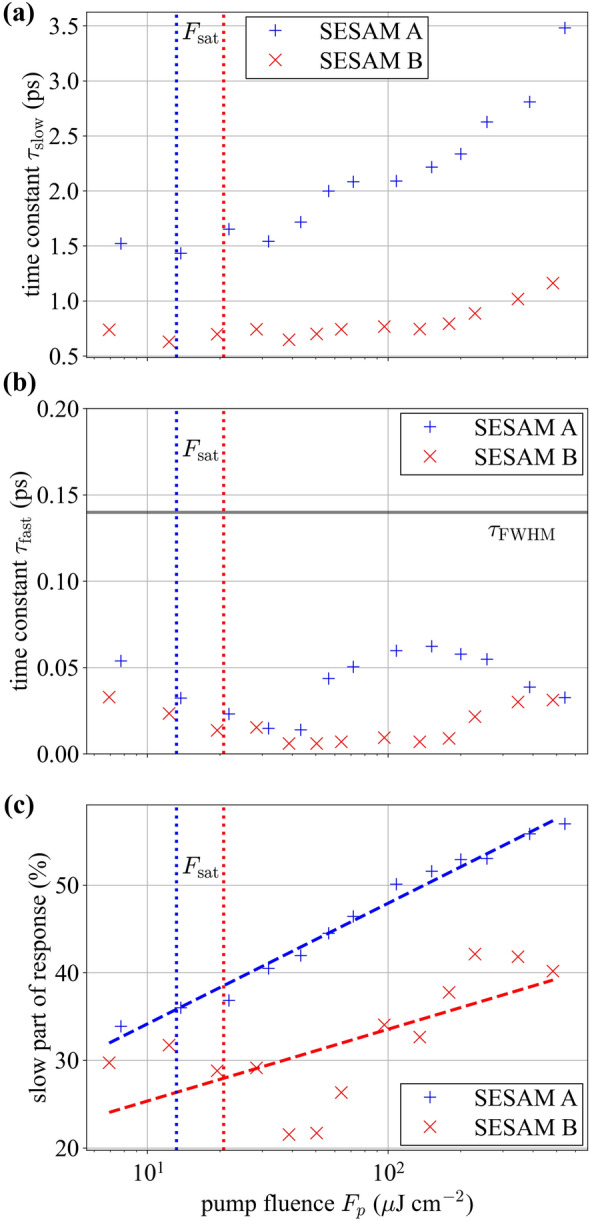
Extracted temporal fit parameters versus pump fluence for SESAMs A and B. **a** The slow and **b** the fast response of the sample. The horizontal reference line marked *τ*_FWHM_ indicates the pulse duration of the pump pulses used for the ETS measurement. **c** The fraction of the full response that corresponds to the slow recovery timescale. The dashed lines are linear fits for the logarithm of the fluence (included as a guide to the eye) and the vertical dotted lines indicate the saturation fluence

The fit parameters from the model provide a convenient means to quantify the changes in the ultrafast recovery dynamics of the SESAMs. Plots of these parameters versus fluence are shown in Fig. 6a–c, where the dashed lines in (c) are a linear least-squares fit included as a guide to the eye. The slow recovery coefficient τ_slow_ increases with fluence (see Fig. 6a), as does the relative contribution of this slow part of the response for SESAM A (see Fig. 6c). As discussed above, the fast recovery coefficient is consistently much faster than the pulse duration and hence the true value is below the resolution of the measurement. It is not our goal here to understand the physical origin of these trends, but rather to illustrate the scale of such changes versus fluence (of some tens of %) and hence the scale of errors that might be incurred when measuring devices at a different fluence to the one at which it will be used in a laser. Soliton modelocked lasers should usually be insensitive to the scale of changes shown here since there is quite a substantial range of recovery times that support stable modelocked operation [22].

### Nonlinear reflectivity characteristics

**Fig. 7:**
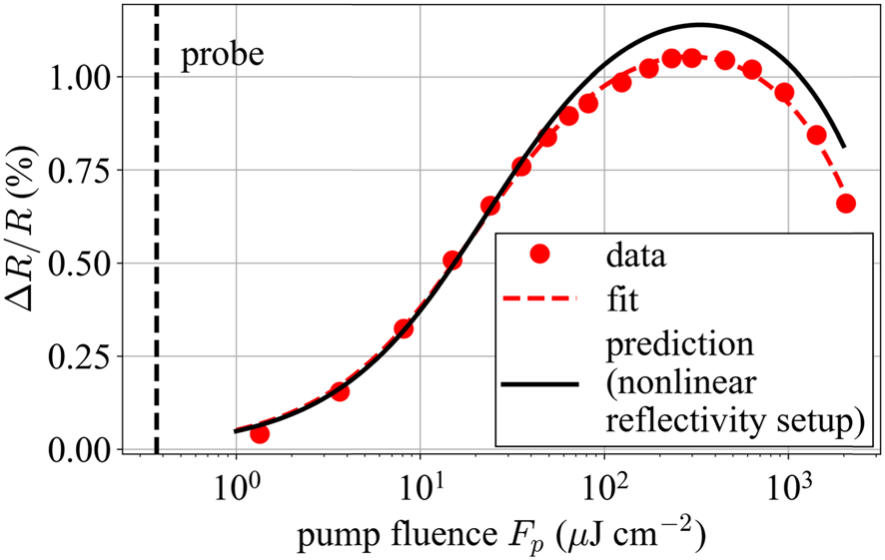
Plot of normalized reflectivity Δ*R*/*R* against pump fluence for sample A. The value of Δ*R*/*R* is taken at the time of temporal overlap of the pump and probe on the sample. The red dashed line is the fit of the model through the data (Eq. (4)). The solid black line corresponds to a simulation of Eq. (4) using the same probe pulse as the ETS measurement and the fit parameters determined from an independent measurement with the nonlinear reflectivity setup

Next, we consider the nonlinear reflectivity characteristics provided by the ETS measurements of sample A. By taking the reflectivity at the zero-delay times of the ETS traces and plotting them versus fluence, one can obtain a plot analogous to a single-pulse nonlinear reflectivity measurement, as seen in Fig. 7. We fit these data to the model as expressed in Eq. (4) (dashed curve). For comparison, the solid black line is obtained using Eq. (4) but with the parameters as measured by the single-pulse measurement to predict the curve for a probe as used in the ETS measurement. To make this comparison, the *F*_2_ parameter inferred from the single-pulse measurement (which uses pulses of duration 183 fs) has been scaled by (141/183) to estimate its value for a pulse duration of 141 fs (as used for the ETS data). This correction is valid for a two-photon absorption-based rollover mechanism; the true mechanism may include other effects as well, so this is only an approximation. Nonetheless, Fig. 7 shows that when applying the *F*_2_ correction factor and accounting for the (minor) influence of the probe fluence (whose value is indicated by the dashed vertical line), there is excellent agreement between the ETS measurements and the adjusted single-pulse nonlinear reflectivity measurements. The fit parameters for the two measurements are given in Table 1.

**Table 1:** Summary of the fit parameters from the two measurement setups

Measurement	Δ*R* (%)	Δ*R*_ns_ (%)	*F*_sat_ (μJ/cm^2^)	*F*_2_ (mJ/cm^2^)
Single-pulse	1.36	0.12	13.2	433
ETS	1.28	–	11.7	395

A potential source of error in the measurements comes from the beam size measurement for the fluence. This is a combination of the uncertainty when placing the beam-profiler (BR2-IGA, DataRay Inc.) in the focus as well as the uncertainty when placing the sample [47]. We estimate an error of 10% for the combined uncertainty. Although *F*_sat_ and *F*_2_ are mainly affected by this error, Δ*R* is also affected during the fitting process. The main uncertainty affecting Δ*R* is the time zero calibration. The error on Δ*R* is estimated to be approximately 5% based on the repeatability of the measurements. Modelocked lasers can usually tolerate significantly more than 5% relative variation in the modulation depth, saturation fluence and *F*_2_ parameters (e.g., 1.05% modulation depth instead of 1%). Therefore, the small uncertainties in our measurements imply that the ETS setup is sufficient for practical characterization of the SESAM’s nonlinear reflectivity as well as its recovery time from a single setup.

### Measurement considerations for inverse saturable absorption

Next, we consider the influence of the ETS setup architecture on the measurements and the information thereby obtained. A significant difference between our setup and the one of [35] is that we use a non-collinear beam geometry and have full control over the individual pump and probe beam polarization, whereas their measurements used a collinear setup and cross-polarized beams. The latter is beneficial for achieving optimal overlap between the beams and sampling the AOI = 0° response, but there is no option for co-polarized beams while keeping the pump and probe beams distinguishable. Since nonlinear processes often depend on polarization, we decided to investigate the dependence of the nonlinear reflectivity on polarization.

**Fig. 8:**
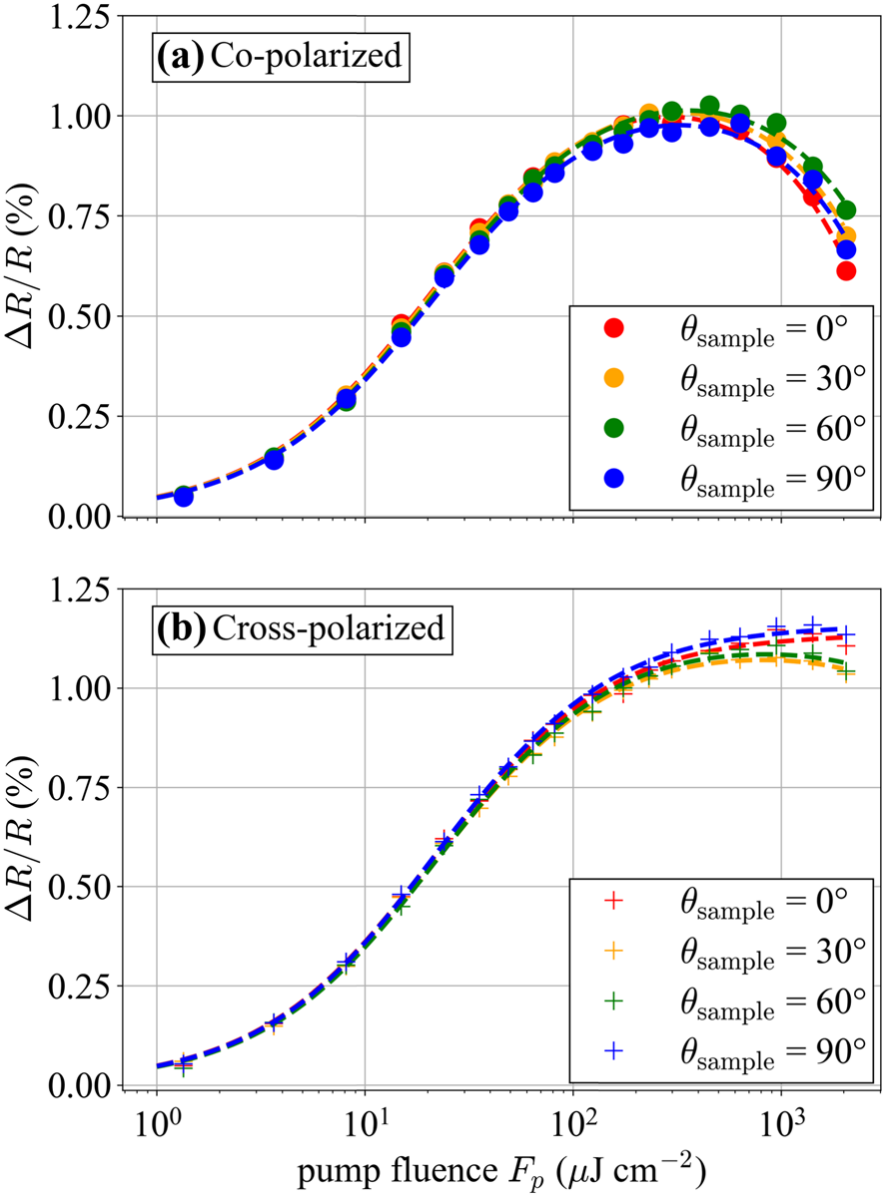
Plots of probe reflectivity versus pump fluence for two configurations of the pump and probe polarization states: **a** co-polarized and **b** cross-polarized. In each configuration, we measure for several rotation angles θ_sample_ of the SESAM around the beam axis. As can be seen, the inverse saturable absorption is much less pronounced in the cross-polarized configuration compared to the co-polarized configuration. Furthermore, there is a dependence on the relative angle between the pump polarization and the rotation angle of the SESAM. The dashed lines are fits of the data to guide the eye

**Fig. 9:**
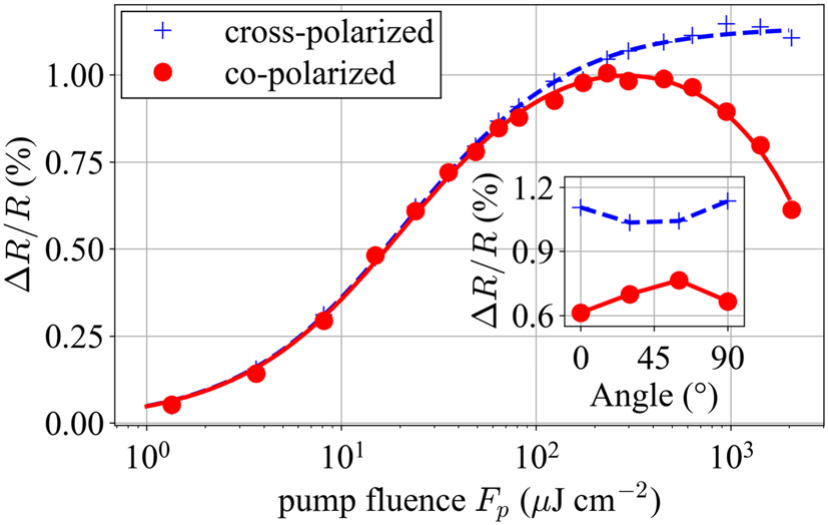
Plot of Δ*R*/*R* versus fluence for the sample at 0° angle for cross- and co-polarized pump and probe beams. There is a clear difference in inverse saturable absorption between the two polarization configurations. The inset shows a plot of Δ*R*/*R* at delay *τ* = 0 versus angle θ_sample_ for the highest fluence measured (*F*_p_ = 2.06 mJ/cm^2^)

It has been shown that two-photon absorption is anisotropic in GaAs [48], as well as in quantum well structures [49]. Moreover, as discussed in [48] a probe beam can experience a larger nonlinear absorption coefficient, in analogy with the relative scaling between cross- and self-phase modulation [50]. Therefore, it can be expected that these effects will influence the *F*_2_ parameter in the measurement. To study this, we performed ETS measurements on sample A for both the co- and cross-polarized case for several different rotation angles of the sample around the beam axis. Figure 8 shows the resulting ETS measurement at four angles (0°, 30°, 60°, 90°). The rollover is noticeably different for the two polarization configurations, and there is a small but clear dependence on the rotation angle of the sample. To show these dependencies more clearly, in Fig. 9, we plot the co- and cross-polarized measurements for a sample angle θ_sample_ = 0°, and in the inset, we show the reflectivity at zero delay and the highest fluence value (*F*_p_ = 2.06 mJ/cm^2^) as a function of sample rotation angle. This figure reveals several interesting features: the rollover is much stronger for the co-polarized case (the *F*_2_ parameter is approximately a sixth of that of the cross-polarized case); it depends on rotation of the sample around the beam axis; and the dependence on this angle is opposite for the two polarization configurations. Based on these observations and the characteristics of two-beam two-photon absorption that have been discussed in the literature [48], we may conclude that neither our setup nor the one of [35] can be expected to perfectly reproduce a single-pulse nonlinear reflectivity measurement due to the non-equivalence of the underlying physics. Further work, including a physical model of the processes involved, would be needed to clarify this question. Nonetheless, the surprisingly good agreement we observe is sufficient from a practical laser design point of view and the fitted *F*_2_ parameter in the co-polarized case is close to that found from the single-pulse nonlinear reflectivity measurement.

## Conclusions

Given their extensive usage in ultrafast laser development, precise characterization of SESAMs remains an important task. Yet doing so can be quite cumbersome due to the need for a pump-probe setup with a mechanical delay line, in combination with a second setup for nonlinear reflectivity measurements. Here, we demonstrate complete characterization of the nonlinear reflectivity and recovery time parameters of SESAMs from a single setup via equivalent time sampling. The new setup is in good quantitative agreement with nonlinear reflectivity measurements. To further simplify the system, we have used a novel dual-comb laser system which provides high pulse energy and a fast sweep of the optical delay between the two combs [20]. Hence, all parameters can be obtained from a single setup at high speed, sensitivity, and precision with no moving parts except the rotation of waveplates and no complex feedback loops to the laser.

We studied two low-temperature-grown GaAs-based samples suitable for modelocking of femtosecond lasers. After validating that the nonlinear reflectivity properties are recovered with high accuracy, we investigated the dynamics of the SESAMs and their dependence on fluence. The temporal response can be captured well by a rate equation based model with two time constants. The model only breaks down at very high fluences in the rollover regime. By fitting the data with this differential equation model, we extract the time constants of the SESAMs at different values of fluence, and observe a gradual increase in the response time at higher fluence. Nonetheless, the response still stays in the few-picosecond regime for both samples. Since our setup uses non-collinear beams, in contrast to [35] which used cross-polarized beams, we could investigate the polarization dependence of the SESAM response. We find that rollover effects are greatly reduced with cross-polarized beams and also depend on the orientation of the sample. These dependencies may originate from the structure of the third-order nonlinearity tensor in the sample. Although a non-collinear setup has fundamental differences to a single-beam setup as used in [34], our results show that the SESAM parameters, including the rollover parameter *F*_2_, are recovered with sufficient accuracy for practical laser design purposes.

While we did not do so here, a calibration measurement with a highly reflective mirror could be performed to characterize SESAMs for fiber lasers where the linear reflectivity is typically far lower. The same setup could also be used to investigate other semiconductor samples including vertical external cavity surface emitting laser (VECSEL) chips or optoelectronic devices, since recovery times up to 12.5 ns can be measured. Our results, therefore, represent a versatile toolset for ultrafast laser development.

## References

[1] Garside BK, Lim TK (1973). J. Appl. Phys..

[2] Valdmanis J, Fork R (1986). IEEE J. Quantum Electron..

[3] Keller U, Miller DB, Boyd GD, Chiu TH, Ferguson JF, Asom MT (1992). Opt. Lett..

[4] Keller U, Weingarten KJ, Kärtner FX, Kopf D, Braun B, Jung ID, Fluck R, Hönninger C, Matuschek N, Aus der Au J (1996). IEEE J. Sel. Top. Quantum Electron..

[5] Keller U (2003). Nature.

[6] Siegman AE (1986). Lasers.

[7] Hönninger C, Paschotta R, Morier-Genoud F, Moser M, Keller U (1999). JOSA B.

[8] Sutter DH, Steinmeyer G, Gallmann L, Matuschek N, Morier-Genoud F, Keller U, Scheuer V, Angelow G, Tschudi T (1999). Opt. Lett..

[9] Spühler GJ, Paschotta R, Fluck R, Braun B, Moser M, Zhang G, Gini E, Keller U (1999). J. Opt. Soc. Am. B.

[10] Spence DE, Kean PN, Sibbett W (1991). Opt. Lett..

[11] Ell R, Morgner U, Kärtner FX, Fujimoto JG, Ippen EP, Scheuer V, Angelow G, Tschudi T, Lederer MJ, Boiko A, Luther-Davies B (2001). Opt. Lett..

[12] Yefet S, Pe’er A (2013). Appl. Sci..

[13] Mayer AS, Phillips CR, Keller U (2017). Nat. Commun..

[14] Shoji TD, Xie W, Silverman KL, Feldman A, Harvey T, Mirin RP, Schibli TR (2016). Optica.

[15] Maas DJHC, Bellancourt A-R, Rudin B, Golling M, Unold HJ, Südmeyer T, Keller U (2007). Appl. Phys. B.

[16] Alfieri CGE, Waldburger D, Nürnberg J, Golling M, Keller U (2019). Opt. Lett..

[17] Barh A, Heidrich J, Alaydin BO, Gaulke M, Golling M, Phillips CR, Keller U (2021). Opt. Express.

[18] Link SM, Klenner A, Mangold M, Zaugg CA, Golling M, Tilma BW, Keller U (2015). Opt. Express.

[19] Willenberg B, Pupeikis J, Krüger LM, Koch F, Phillips CR, Keller U (2020). Opt. Express.

[20] Pupeikis J, Willenberg B, Bruno F, Hettich M, Nussbaum-Lapping A, Golling M, Bauer CP, Camenzind SL, Benayad A, Camy P, Audoin B, Phillips CR, Keller U (2021). Opt. Express.

[21] Saltarelli F, Graumann IJ, Lang L, Bauer D, Phillips CR, Keller U (2019). Opt. Express.

[22] Kärtner FX, Jung ID, Keller U (1996). IEEE J. Sel. Top. Quantum Electron..

[23] Shah J (1999). Ultrafast spectroscopy of semiconductors and semiconductor nanostructures.

[24] Liu X, Prasad A, Chen WM, Kurpiewski A, Stoschek A, Liliental-Weber Z, Weber ER (1994). Appl. Phys. Lett..

[25] Smith FW, Calawa AR, Chen C-L, Manfra MJ, Mahoney LJ (1988). IEEE Electron Device Lett..

[26] Witt GL, Calawa R, Mishra U, Weber E (1992). Low Temperature (LT) GaAs and Related Materials.

[27] Johnson MB, McGill TC, Paulter NG (1989). Appl. Phys. Lett..

[28] Wood CD, Hatem O, Cunningham JE, Linfield EH, Davies AG, Cannard PJ, Robertson MJ, Moodie DG (2010). Appl. Phys. Lett..

[29] Burford NM, El-Shenawee MO (2017). Opt. Eng..

[30] Kilen I, Hader J, Moloney JV, Koch SW (2014). Optica.

[31] Grange R, Haiml M, Paschotta R, Spühler GJ, Krainer L, Golling M, Ostinelli O, Keller U (2005). Appl. Phys. B.

[32] Haiml M, Grange R, Keller U (2004). Appl. Phys. B Lasers Opt..

[33] Maas DJHC, Bellancourt A-R, Hoffmann M, Rudin B, Barbarin Y, Golling M, Südmeyer T, Keller U (2008). Opt. Express.

[34] Maas DJHC, Rudin B, Bellancourt A-R, Iwaniuk D, Marchese SV, Südmeyer T, Keller U (2008). Opt. Express.

[35] Fleischhaker R, Krauß N, Schättiger F, Dekorsy T (2013). Opt. Express.

[36] Weingarten KJ, Rodwell MJW, Heinrich HK, Kolner BH, Bloom DM (1985). Electron. Lett..

[37] Weingarten KJ, Rodwel MJW, Bloom DM (1988). IEEE J. Quantum Electron..

[38] Elzinga PA, Kneisler RJ, Lytle FE, Jiang Y, King GB, Laurendeau NM (1987). Appl. Opt..

[39] Schiller S (2002). Opt. Lett..

[40] Coddington I, Newbury N, Swann W (2016). Optica.

[41] Saraceno CJ, Schriber C, Mangold M, Hoffmann M, Heckl OH, Baer CRE, Golling M, Südmeyer T, Keller U (2012). IEEE J. Sel. Top. Quantum Electron..

[42] Diebold A, Zengerle T, Alfieri CGE, Schriber C, Emaury F, Mangold M, Hoffmann M, Saraceno CJ, Golling M, Follman D, Cole GD, Aspelmeyer M, Südmeyer T, Keller U (2016). Opt. Express.

[43] Lang L, Saltarelli F, Lacaille G, Rowan S, Hough J, Graumann IJ, Phillips CR, Keller U (2021). Opt. Express.

[44] Schoenlein RW, Lin WZ, Ippen EP, Fujimoto JG (1987). Appl. Phys. Lett..

[45] Agrawal GP, Olsson NA (1989). IEEE J. Quantum Electron..

[46] Sieber OD, Hoffmann M, Wittwer VJ, Mangold M, Golling M, Tilma BW, Südmeyer T, Keller U (2013). Appl. Phys. B.

[47] Orsila L, Härkönen A, Hyyti J, Guina M, Steinmeyer G (2014). Opt. Lett..

[48] Dvorak MD, Schroeder WA, Andersen DR, Smirl AL, Wherrett BS (1994). IEEE J. Quantum Electron..

[49] Le HQ, Di Cecca S (1991). Opt. Lett..

[50] Agrawal GP (2013). Nonlinear Fiber Optics.

